# Screening of Leafy Vegetable Varieties with Low Lead and Cadmium Accumulation Based on Foliar Uptake

**DOI:** 10.3390/life12030339

**Published:** 2022-02-24

**Authors:** Zhangqian Xu, Jianwei Peng, Zhen Zhu, Pengyue Yu, Maodi Wang, Zhi Huang, Ying Huang, Zhaojun Li

**Affiliations:** 1 National Engineering Research Center for Efficient Utilization of Soil and Fertilizer Resources, College of Resources and Environment, Hunan Agricultural University, Changsha 410128, China; xuzhangqian@stu.hunau.edu.cn (Z.X.); 18873303786@stu.hunau.edu.cn (Z.Z.); yupengyue@stu.hunau.edu.cn (P.Y.); sx20210226@stu.hunau.edu.cn (M.W.); 18714838265@stu.hunau.edu.cn (Z.H.); 2 Key Laboratory of Plant Nutrition and Fertilizer, Ministry of Agriculture and Rural Affairs, Institute of Agricultural Resources and Regional Planning, Chinese Academy of Agricultural Sciences, Beijing 100081, China; lizhaojun@caas.cn

**Keywords:** lead (Pb), cadmium (Cd), leafy vegetable, foliar uptake, varieties screening

## Abstract

Leafy vegetables cultivated in kitchen gardens and suburban areas often accumulate excessive amounts of heavy metals and pose a threat to human health. For this reason, plenty of studies have focused on low accumulation variety screening. However, identifying specific leafy vegetable varieties according to the foliar uptake of air pollution remains to be explored (despite foliar uptake being an important pathway for heavy-metal accumulation). Therefore, in this study, the lead (Pb) and cadmium (Cd) contents, leaf morphology, and particle matter contents were analyzed in a micro-area experiment using 20 common vegetables. The results show that the Pb content in leaves ranged from 0.70 to 3.86 mg kg^−1^, and the Cd content ranged from 0.21 to 0.99 mg kg^−1^. Atmospheric particles were clearly scattered on the leaf surface, and the particles were smaller than the stomata. Considering the Pb and Cd contents in the leaves and roots, stomata width-to-length ratio, leaf area size, enrichment factor, and translocation factor, Yidianhongxiancai, Qingxiancai, Baiyuanyexiancai, Nanjingjiangengbai and Sijixiaobaicai were recommended for planting in kitchen gardens and suburban areas as they have low accumulation characteristics. Identifying the influencing factors in the accumulation of heavy metals in vegetables through foliar uptake is important to help plant physiologists/environmentalists/policy makers to select suitable varieties for planting in air-polluted areas and thus reduce their threat to human health.

## 1. Introduction

Heavy-metal pollution in farmlands has attracted a great deal of attention in China, especially in relation to the potential impact on human health caused by the intake of heavy-metal-contaminated crops. Among all crop varieties, heavy-metal overaccumulation is particularly significant in vegetables and rice. The term “lead vegetables” is often used in China to describe heavily lead-contaminated vegetables, as is the term “cadmium rice” [1]. Lead (Pb) and cadmium (Cd) are considered to be two of the most toxic elements to human health. Long-term high dose exposure to Pb has adverse effects on blood enzymes and the central nervous system [2]. Chronic exposure to Cd was reported to cause pulmonary adenocarcinomas, lung cancer, kidney dysfunction, and bone fractures [3]. According to the 2016 Dietary Guidelines for Chinese, at least one third of foods are vegetables, which represents up to 500–850 g per meal [4]. Industrial processes such as smelting, e-waste processing, coal combustion, waste incineration, vehicular traffic, pesticide use, and fertilization contribute to increased Pb and Cd concentrations in the environment [5,6]. Moreover, the majority of consumed vegetables originate from kitchen gardens located in peri-urban areas [7,8], which are generally close to multiple pollution sources resulting from human pressure, traffic emission, urban waste disposal, etc. [9]. Various studies have been conducted in these areas to assess soil and vegetable contamination [10,11]. Daimari et al. (2020) assessed trace metal air pollutants in urban, peri-urban, and rural areas on the Brahmaputra Valley plain and found that metals related to automobiles were accumulated in greater volume in samples from peri-urban locations [12]. For example, Chen et al. (2018b) and Li et al. (2015) discovered that Pb and Cd contents in vegetables were 0.04–0.72 mg kg^−1^ and 0.02–0.63 mg kg^−1^, respectively, which exceeded the national standards (GB2762-2017) [13,14]. Huang et al. (2018) analyzed the heavy-metal contents in vegetables in a peri-urban area in Zhejiang province and showed the hazard index caused by the intake of local heavy-metal-containing vegetables was significantly higher than 1, indicating significant adverse health effects on local residents [11]. Therefore, exploring the uptake and translocation of heavy metals in vegetables is crucial for maintaining food safety and human health.

The existing studies mainly focus on heavy-metal uptake from soil by roots and explore the mechanisms involved in root uptake, translocation, and detoxification [15]. On this basis, a multitude of studies focus on screening for low accumulation varieties, which can be promoted in order to maintain food safety. Wei et al. (2017) planted 20 pakchoi genotypes in soils with both low and high levels of Cd and As co-contamination soils in order to identify low health risk genotypes [16]. Liu et al. (2009) compared the translocation factor (TF) and enrichment factor (EF) among 40 cabbage genotypes, and concluded that only Lvxing 70 could be regarded as a Cd-excluder genotype [17]. Moreover, various researchers suggest that the correlation between heavy-metal accumulation in vegetables and the corresponding soil is weak, whereas there is a direct correlation with the amount of heavy metals in atmospheric depositions [18,19]. Bi et al. (2018) conducted a Pb isotope ratio analysis on 48 road dusts and 106 leafy vegetable samples collected in Shanghai Industrial Park and found that the accumulation of Pb in leafy vegetables was directly derived from atmospheric deposition [20]. Another study demonstrated that the main source of Pb in cabbage leaves was local PM_2.5_ [21]. He et al. (2021) suggested that atmospheric deposition in certain areas has a great impact on the concentration of heavy metals in crop leaves, and the influencing factors include cuticle, lenticel, and stomata structure, etc [22]. Overall, atmospheric deposition has been shown to be an important source of heavy-metal accumulation in leafy vegetables. However, knowledge regarding the mechanism of foliar uptake, translocation, and accumulation is limited [23]. In addition, no species have been identified for cultivation in air-polluted areas based on studies of foliar uptake.

This led is to hypothesize that the accumulation of heavy metals in vegetables may be due to both root uptake from soil and foliar uptake from the atmosphere, especially for leafy vegetables. Therefore, in this study, the Pb and Cd contents in 20 common vegetables (water spinach (*Ipomoea aquatica Forssk*), amaranth (*Amaranthus tricolor*), cabbage (*Brassica pekinensis*)), and the particulate matter content and leaf morphology were analyzed in a micro-area experiment with the aim of: (1) identifying the degree of Cd and Pb accumulation in atmospheric-plant systems and their risks to the population; (2) exploring the differences in foliar uptake and influencing factors among these varieties; and (3) recommending suitable varieties based on foliar uptake characteristics to maintain food safety.

## 2. Material and Methods

### 2.1. Site Description and Soil Characterization

The study was conducted in the experimental base of Hunan Agricultural University Changsha City, southern China (28°11′12″ N, 113°5′29″ E, altitude 43 m). This area is in a suburban area and beside a city road. The airborne Pb and Cd originated from natural atmospheric deposition, which was mainly emitted from traffic. The leading wind direction in the area is primarily southeasterly in summer and northeasterly in winter. The annual average temperature is 16–18 °C, with an annual average humidity of 70%–80%. The average annual precipitation ranges from 1400 to 1700 mm. Three species of 20 common edible vegetables (Table 1)—water spinach (*Ipomoea aquatica Forssk*, 3 varieties), amaranth (*Amaranthus tricolor*, 5 varieties), and cabbage (*Brassica pekinensis*, 12 varieties)—were selected and the treatment was repeated three times. Each vegetable was randomly planted in a 2 m × 2 m field. Vegetable seeds were sown on 2 June 2019 and harvested on 2 September 2019. The basic physical and chemical properties of the soil that we analyzed were organic matter (11.6 ± 0.78 g kg^−1^), pH (8.08 ± 0.06), total nitrogen (0.82 ± 0.20 g kg^−1^), phosphorus (484 ± 0.18 mg kg^−1^), potassium (17.4 ± 0.11 g kg^−1^), total Pb (24.31 ± 0.19 mg kg^−1^), and total Cd (0.14 ± 0.04 mg kg^−1^).

### 2.2. Sample Collection and Analysis

#### 2.2.1. Particulate Matter Samples

The particulate matter samples (PM10 and TSP) were collected by two automatic precipitation and dust fall samplers each day from June 2019 to August 2019. The airflow rate was 28 L min^−1^ with 50% cutoff sizes of <10.0 µm (PM10) and total suspended particles (TSP). The particulate matter samples were digested using an HNO_3_-HCl (3:1) mixture at 105 ± 5 °C for 2 h, cooled to room temperature, and diluted with deionized water to 30 mL. The aqueous samples were first acidified with sub-boiling quartz distilled 6 M HCl, and then digested using 2 mL HNO_3_ (1:1) and 1 mL HCl (1:1) at 85 °C until the sample evaporated to 20 mL. Sample replicates and reagent blanks were included in each batch of analysis to ensure the quality of the analysis.

#### 2.2.2. Vegetable and Soil Samples

Vegetable samples were collected at the maturity stage using a crossover method and the samples were rinsed thoroughly with deionized water to remove any attached soil/substrate particles before being separated into shoots and roots. At the same time, surface soil (0–20 cm) samples were collected from each plot. The soil samples were dried at room temperature and homogenized using a mortar and pestle. One portion of the sample was passed through 2 mm sieves to measure the pH content, and another was passed through 0.145 mm sieves to determine the Pb and Cd contents. Subsamples of shoots and roots were firstly dried in oven at 105 °C for 30 min and then stored in oven at 80 °C until reaching a constant weight. They were then crushed with prototype powder. Soil samples were digested with a mixture of HNO_3_-H_2_O_2_-HF (6:3:3), and plant materials were digested with HNO_3_-H_2_O_2_ (2:1). The digested solutions were diluted to 25 mL with 1% HNO_3_ solution. All of the samples were stored in the dark at 4 °C and were determined within one month. To ensure the reliability and quality of the data, standard reference soil (GBW07387) and bush twigs and leaves (GBW07602) from the Center of National Reference Materials of China were measured together with soil and plant samples.

The Pb and Cd concentrations in solutions were determined using an inductively coupled plasma mass spectrometer (ICP-MS, Model NEXION 350 X, PerkinElmer, America). The recovery of spiked standard for each element ranged between 80% and 120% and the detection limits were 0.01 µg mL^−1^ and 0.01 µg mL^−1^ for Pb and Cd, respectively.

### 2.3. Scanning Electron Microscopy (SEM) Analysis

Vegetable leaves were observed using a scanning electron microscope (SEM), as described previously [24]. Small strips of leaf (about 0.5 cm^2^) were trimmed from the area between the margin and mid-rib. The small strips were first stored in 2.5% glutaraldehyde solution overnight for fixation (prefixation), and then in osmium tetroxide for post-fixation for 1 h. After being washed twice with buffer solution for 15 min, these samples were passed through a series of acetone solutions (30%, 50%, 70%, 95% and 100%) for dehydration. Then, they were dried in a critical point drier (CPD) with CO_2_ as the carrier gas. The SEM was carried out using a 30 keV, JEOL JSM-6490 LV scanning microscope (JEOL, China) with standard automated features such as auto focus/stigmator, auto gun, and auto-contrast with multiple live image display. The samples were coated with carbon using a high vacuum system to wet specimens, the upper surfaces of the leaf segments were studied, and micrographs were taken at various magnifications.

### 2.4. Foliar Parameters Analysis

#### 2.4.1. Stomata Size

Bu et al. (2014) used width-to-length ratio to represent the degree of stomatal opening and closing [25]. The stomata sizes were expressed by measuring the long and short axis of the stomata map taken using a scanning electron microscope.

#### 2.4.2. Leaf Surface Area

According to the principle of the mass density formula, a piece of uniform quality paper (normal printing paper is fine) was selected, the rectangle was divided, the length and width was recorded, its area (length × width) was calculated, and then it was weighed. Thereafter, the outline of the leaf was drawn on the paper, it was cut out, and then it was weighed. The leaf area was calculated by dividing the weight of the leaf contour paper by the weight of the entire paper and multiplying this by the area of the entire paper [26]. When measuring quality, five repetitions were used to reduce errors.

### 2.5. Health Risk Assessment

The health risk from heavy metals are mainly derived from three pathways: the ingestion of the contaminated crops, inhalation, and dermal contact with the polluted particulate matter. The health risk can be estimated using the ratio of the estimated daily dose (EDD, (mg kg^−1^ day^−1^)) to the reference dose (RfD, (mg kg^−1^ day^−1^)). If the EDD is lower than the RfD, there are considered to be no health risk. Reference doses were based on 0.004 and 1 × 10^−3^ mg kg^−1^ day^−1^ for Pb and Cd, respectively [27,28]. The estimated daily dose of Pb and Cd by consuming leaves of leafy vegetables were calculated using the following equation [29]:

(1) $$\mathrm{EDD}=\frac{C_{\mathrm{TMetal}\;}{\times \;\mathrm{IR}}_{\mathrm{veg}}\;\times \;\mathrm{Cf}\;\times \;\mathrm{EF}\;\times \;\mathrm{ED}}{\mathrm{LE}\;\times \;\mathrm{BW}}$$

where ${\mathrm{IR}}_{\mathrm{veg}}$, Cf, EF, ED, LE, and BW represent the ingestion rate of leafy vegetable (100.4 g day^−1^ for adults, 66.9 g day^−1^ for children), conversion factor (0.085 mg kg^−1^), exposure frequency (365 days year^−1^), exposure duration (70 years), life expectancy (25,550 days), and average body weight (70 kg for adults, 24.5 kg for children), respectively [30].

### 2.6. Enrichment Factor

The enrichment factor (EF) was used to differentiate between natural and anthropogenic sources. It was calculated using the following equation [31]:

(2) $$\mathrm{EF}=\frac{{\left(C_{\mathrm{TMetal}}{/C}_{\mathrm{ref}}\right)}_{\mathrm{sample}}}{{\left(C_{\mathrm{TMetal}}{/C}_{\mathrm{ref}}\right)}_{\mathrm{background}}}$$

where $C_{\mathrm{ref}}$ represents the reference element concentration (mg kg^−1^ for soil, ug m^−3^ for air). Fe was selected as a reference element for geochemical normalization due to its uniform natural concentration and natural abundance in the Earth’s crust [32].

### 2.7. Statistical Analysis

All data analyses were performed using SPSS 16.0 and Origin Pro 8.0. Variance analysis was subjected to a factorial analysis of variance (ANOVA) using a least significant difference (LSD) test at a significance level of *p* < 0.05 and *p* < 0.01. The cluster heat map showed the row and column hierarchy of clusters in the data matrix. The heat map of distribution profiles and physiological and chemical indicators among the 20 vegetables were generated in RStudio with the ggplot2 package [33].

## 3. Results

### 3.1. The Concentration of Pb and Cd in Atmospheric Particulate Matter

**Figure 1:**
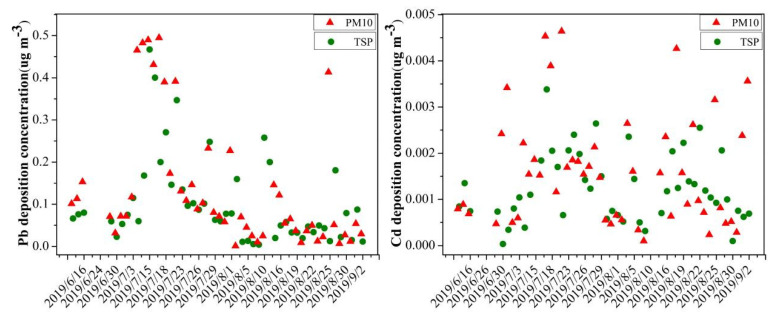
The concentration of Pb and Cd in atmospheric particulate matter (TSP and PM10) during the entire vegetable growing period.

It can be seen that the concentrations varied greatly at different times. During the test days, the average concentration of Pb in TSP and PM10 was 0.1602 (0.0224–0.4936) µg m^−3^ day^−1^ and 0.2050 (0.0568–0.4815) µg m^−3^ day^−1^, respectively (Figure 1). The concentration of Cd in TSP and PM10 was 0.0022 (0.001–0.0244) µg m^−3^ day^−1^ and 0.0017 (0.001–0.0046) µg m^−3^ day^−1^, respectively. As compared with the first level of the ambient air quality standard (GB3095-2012, 0.5 µg m^−3^ day^−1^), the concentrations of TSP-Pb and PM10-Pb were both lower than the standard, and first exhibited an increasing trend and then a decreasing trend from July to August. The concentrations of TSP-Cd and PM10-Cd tended to be more stable and evenly distributed throughout the growth period and did not exceed the secondary ambient air quality standard (GB3095-2012, 0.005 µg m^−3^ day^−1^). In addition, the concentrations of Pb and Cd in PM10 were significantly higher than those in TSP, indicating that Pb and Cd were mainly concentrated in fine particles.

### 3.2. The Concentration of Pb in Roots and Leaves

**Figure 2:**
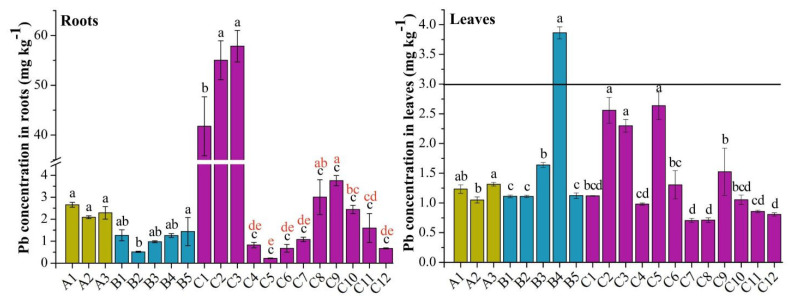
The concentration of Pb in dry-weight roots and leaves in three species of leafy vegetables: A, water spinach (*Ipomoea aquatica Forssk*); B, amaranth (*Amaranthus tricolor*); C, cabbage (*Brassica pekinensis*); the line represents the maximum permissible concentration (MPC) for Pb. The bars represent means ± SD (*n* = 3). Different lowercase letters indicate significant differences at the *p* < 0.05 level of LSD test using spinach, amaranth, cabbage (black lowercase), and nine kinds of cabbage (red lowercase).

The concentrations of Pb in dry-weight roots and leaves are shown in Figure 2. The contents in roots ranged from 0.22 to 57.84 mg kg^−1^, of which C5 had the lowest concentration (0.22 mg kg^−1^), while C3 had the highest (57.84 mg kg^−1^). Overall, the concentration exhibited the following trend: cabbage (14.07 mg kg^−1^) > water spinach (2.34 mg kg^−1^) > amaranth (1.09 mg kg^−1^), and the contents of Pb in roots exhibited the biggest variation among cabbages. The contents in C3 and C2 were significantly higher than C1, and they were significantly higher in these three than in the others cabbage species. Furthermore, there was no significant difference between or among amaranth and water spinach. The contents in leaves ranged from 0.70 to 3.86 mg kg^−1^, of which C7 had the lowest concentration (0.70 mg kg^−1^), while B4 had the highest (3.86 mg kg^−1^). Overall, the concentration exhibited the following trend: amaranth (1.77 mg kg^−1^) > cabbage (1.38 mg kg^−1^) > water spinach (1.20 mg kg^−1^), which was not consistent with Pb in roots. Moreover, the contents of Pb in leaves did not exhibit significant variation among varieties. The accumulation of Pb was biggest in B4, while it was lowest in C12, C8, and C7.

According to limits of contaminants in food (National Standard Agency of China, GB2762-2017), the maximum permissible concentration (MPC) for Pb in leafy vegetables is 0.3 mg kg^−1^ fresh weight. The monitored vegetables contained about 90% water. Therefore, in these 20 varieties, only B4 samples (3.86 mg kg^−1^) exceeded the MPC and posed potential health risks. Moreover, the C3, C2, and C1 cabbage species were close to the MPC.

### 3.3. The Concentration of Cd in Roots and Leaves

**Figure 3:**
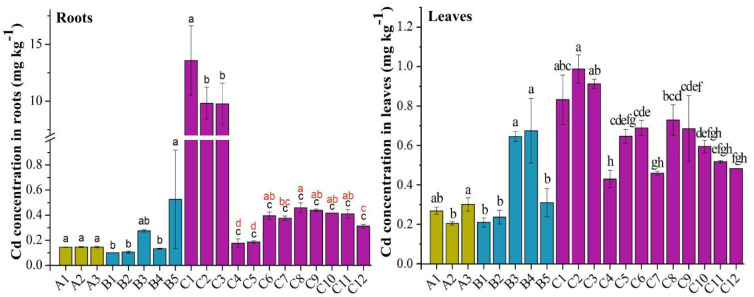
The concentration of Cd in dry-weight roots and leaves: A, water spinach (*Ipomoea aquatica Forssk*); B, amaranth (*Amaranthus tricolor*); C, cabbage (*Brassica pekinensis*). The bars represent means ± SD (*n* = 3). Different lowercase letters indicate significant differences at *p* < 0.05 level of LSD test using spinach, amaranth, cabbage (black lowercase), and nine kinds of cabbage (red lowercase).

The concentration of Cd in dry-weight roots and leaves are shown in Figure 3. The contents ranged from 0.10 to 13.58 mg kg^−1^ in the roots of the 20 varieties, of which C1 had the lowest concentration (0.10 mg kg^−1^), while C3 had the highest (13.58 mg kg^−1^). Overall, the concentration exhibited the following trend: cabbage (0.67 mg kg^−1^) > amaranth (0.42 mg kg^−1^) > water spinach (0.26 mg kg^−1^), and the contents of Cd in roots exhibited the biggest variation among cabbages. C1 was significantly higher than C3 and C2, while these three were significantly higher than the other cabbage species; moreover, C8 was significantly higher than C5 and C4. In addition, there was no significant difference between or among water spinach. B5 was significantly higher than B4, B2, and B1 in amaranth. The contents ranged from 0.21 to 0.99 mg kg^−1^ in leaves of the 20 varieties, of which A2 had the lowest concentration (0.21 mg kg^−1^), while C2 had the highest (0.99 mg kg^−1^). Overall, the concentration exhibited the following trend: cabbage (0.67 mg kg^−1^) > amaranth (0.42 mg kg^−1^) > water spinach (0.26 mg kg^−1^). This was consistent with that for Cd in roots.

According to limits of contaminants in food (National Standard Agency of China, GB2762-2017), the maximum permissible concentration (MPC) of Cd in leafy vegetables is 0.2 mg kg^−1^ fresh weight. The monitored vegetables contained about 90% water. Therefore, the contents of Cd in all 20 varieties did not exceed the standard.

### 3.4. Health Risk Assessment

The estimated daily dose (EDD) describes the contents of a certain element in the body’s daily vegetable intake, expressed as the amount of accumulated contaminants in the human body during daily vegetable intake. It can be seen from Table 2 that the average daily intake of Pb from vegetables by adults and children was higher than Cd. In addition, for both Pb and Cd, children’s daily vegetables Pb/Cd intakes were all higher than those of adults. When considering vegetable varieties, it was found that the trend of Pb/Cd contents varied among different varieties. For Pb, the average daily intake of the three major types of vegetables (water spinach, amaranth, cabbage) were 1.46 × 10^−5^, 2.16 × 10^−5^, and 1.68 × 10^−5^ mg kg^−1^ day^−1^ for adults and 2.78 × 10^−5^, 4.11 × 10^−5^, and 3.20 × 10^−5^ mg kg^−1^ day^−1^ for children, respectively, with the following trend: amaranth> cabbage> water spinach. However, for Cd, the average daily intakes of the three major types of vegetables were 3.17 × 10^−6^, 5.10 × 10^−6^, and 8.14 × 10^−6^ mg kg^−1^ day^−1^ for adults and 6.04 × 10^−6^, 9.71 × 10^−6^, and 1.55 × 10^−5^ mg kg^−1^ day^−1^ for children, respectively, with the following trend: cabbage> amaranth> water spinach. In general, the reference doses for Pb and Cd are 0.004 and 1 × 10^−3^ mg kg^−1^ day^−1^, respectively [27,28]. All samples were below the reference doses, indicating that the intake of vegetables grown at the test site did not pose a significant health risk to humans.

### 3.5. The Enrichment and Translocation Factor of Pb and Cd

**Figure 4:**
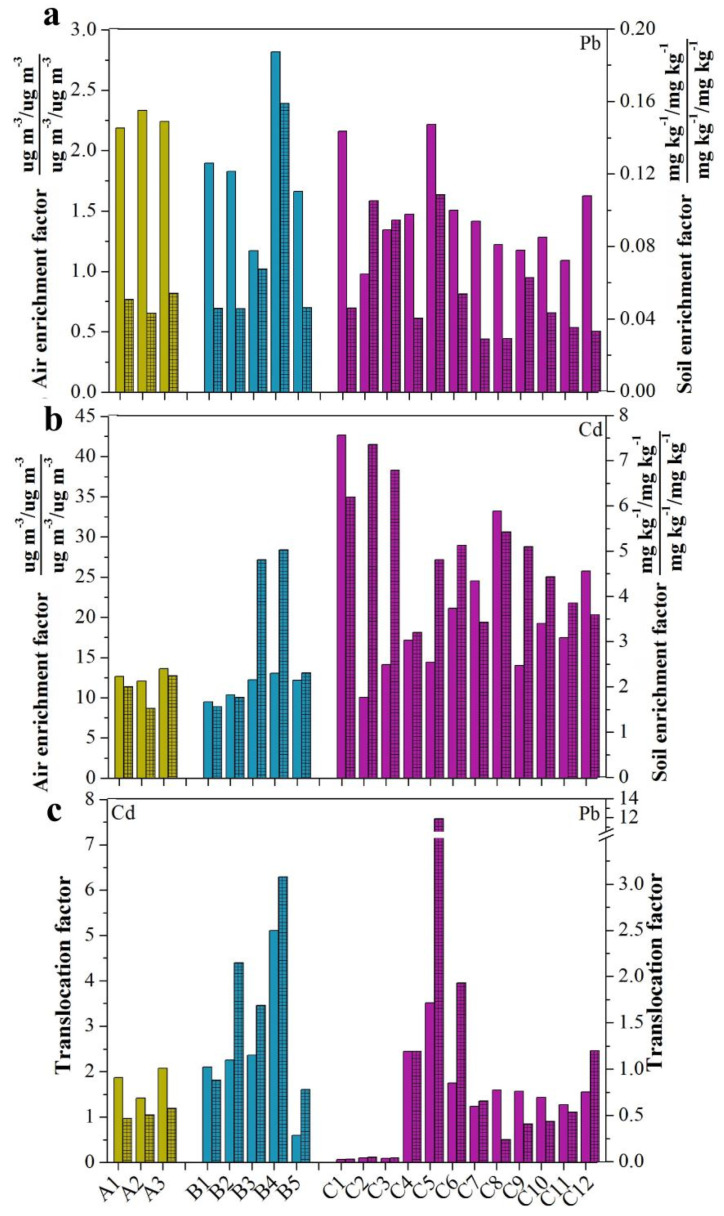
The enrichment and translocation factor of Pb and Cd: A, water spinach (*Ipomoea aquatica Forssk*); B, amaranth (*Amaranthus tricolor*); C, cabbage (*Brassica pekinensis*). (**a**,**b**) the enrichment factor of Pb and Cd in shoots to atmospheric deposition and soil. The unshaded bars represent the air enrichment factors in shoots, and the shaded bars represent the soil enrichment factors in shoots. (**c**) The translocation factor of Pb and Cd from roots to shoots in leafy vegetables. The unshaded bars represent the translocation factors of Cd, and the shaded bars represent the translocation factors of Pb.

The enrichment factor (EF) was calculated as the heavy-metal concentrations in shoots as compared to the concentrations in soil/atmosphere. The EFs of Pb and Cd in shoots as compared to atmospheric deposition and soil are shown in Figure 4a,b. The atmospheric enrichment factors of Pb were basically in the range of 1–2, and the soil enrichment factor was all less than 1 (Figure 4a). Similarly, it can be seen from Figure 4b that the atmospheric enrichment factors of Cd in the 20 leafy vegetables were all higher than 10, and the soil enrichment factors were all less than 10. Overall, the EFs of Pb were lower than those of Cd in the 20 leafy vegetables, and the EFs of shoots compared to the atmosphere were significantly higher than those of the soil. Translocation factor (TF) was calculated as the ratio of the heavy-metal concentration in leaves to that in roots. The TFs of Pb and Cd from roots to shoots in leafy are shown in Figure 4c. It can be seen that the TF of Pb and Cd in the 20 leafy vegetables ranged from 0.03 to 11.86 and from 0.06 to 5.11, respectively. The average TFs of Pb in water spinach, amaranth, and cabbage were 0.51, 1.71, and 1.55, respectively, and similarly, the TFs of Cd were 1.79, 1.39, and 1.58, respectively, exhibiting the following trend: amaranth > cabbage > water spinach. Similarly, with EF, the TF of Pb was also lower than that of Cd. Moreover, only four leafy vegetables, especially the cabbage vegetables, exhibited lower TF of Cd, which was lower than 1.0, while 13 leafy vegetables, including several cabbage vegetables and all water spinach, had lower TF of Pb.

## 4. Discussion

### 4.1. Accumulation and Translocation Characteristics of Pb and Cd among Various Varieties

Enrichment factor (EF), which refers to the content of elements in plants as compared to that in the environment, can be used to evaluate the ability of heavy-metal accumulation [31]. It is widely used in the screening of low heavy-metal accumulation crops and identifying emission sources [34]. Generally, the value is equal to 1.0, indicating that the accumulation of pollutants originates entirely from Earth’s crust or natural weathering processes. However, an EF value greater than 1.5 indicates a considerable volume of trace metal (likely from anthropogenic factors [35]. The EFs of Pb and Cd both exceeded 1.0 (Figure 4a,b), indicating that the accumulation of Pb and Cd generally originated from anthropogenic activities at the experiment site. Moreover, leafy vegetables grown by retail households and cultivated near industrial areas, villages, roads, most of which are exposed to various anthropogenic emissions, likely pose a threat to human health [36]. Luo et al. (2011) found that the EF value measured for Cd in *Allium ascalonicum* L. (leafy vegetables) was 1.258, exhibiting higher a value than *Raphanus sativus* L. and *Daucus carota* L. (non-leafy vegetables) [37]. Therefore, it is necessary to screen leafy vegetables according to heavy-metal accumulation through foliar uptake. According to the EF values, in this study, B1, B2, and C7 had a lower tendency to accumulate Pb and Cd.

Translocation factor (TF), which refers to the content of elements in leaves compared to in roots, can be used to evaluate the ability of heavy-metal translocation. A TF >1.0 indicates preferential partitioning of metals from roots to shoots [38]. Therefore, this study calculated TF to evaluate the capacity of a plant to translocate heavy metals from roots to leaves (Figure 4c) [39]. The TF of Pb and Cd in the 20 leafy vegetables demonstrated obvious variation (Figure 4c). Liu et al. (2009) indicated that an increasing Cd concentration in roots causes evidently decreasing TF values under contaminated soils [17]. Therefore, it can be seen that the TFs of C1, C3, and C2 were significantly lower than those of the other species due to the higher concentrations of Pb and Cd in roots (Figure 2 and Figure 3). Conversely, the lower TFs of A2, B5, and C4 may have resulted from the limited transfer from roots to leaves or foliar uptake. Shahid et al. (2017) demonstrated that differences in physiology, morphology, and the anatomy of each plant, such as leaf inclination angle, branch density, structure of plant canopy, leaf area, and stomata size and density, were major morphological characteristics that affect foliar uptake [40]. Furthermore, the uptake and translocation of trace elements in plants may depend on mobility and competition with other elements [41,42]. Shahid et al. (2020) reported that approximately 90% of Pb was accumulated in the shoot tissues of spinach and that there was limited transfer to roots under foliar treatment of PbO-NPs in leaves [43]. Furthermore, various studies revealed that Pb mobility inside plants was very low, and tended to accumulate near the site of entrance to plants [43,44]. Therefore, the relatively high concentration of Pb in leaves was more likely from foliar uptake.

### 4.2. Effect of Foliar Uptake on Heavy-Metal Accumulation in Leafy Vegetables

**Figure 5:**
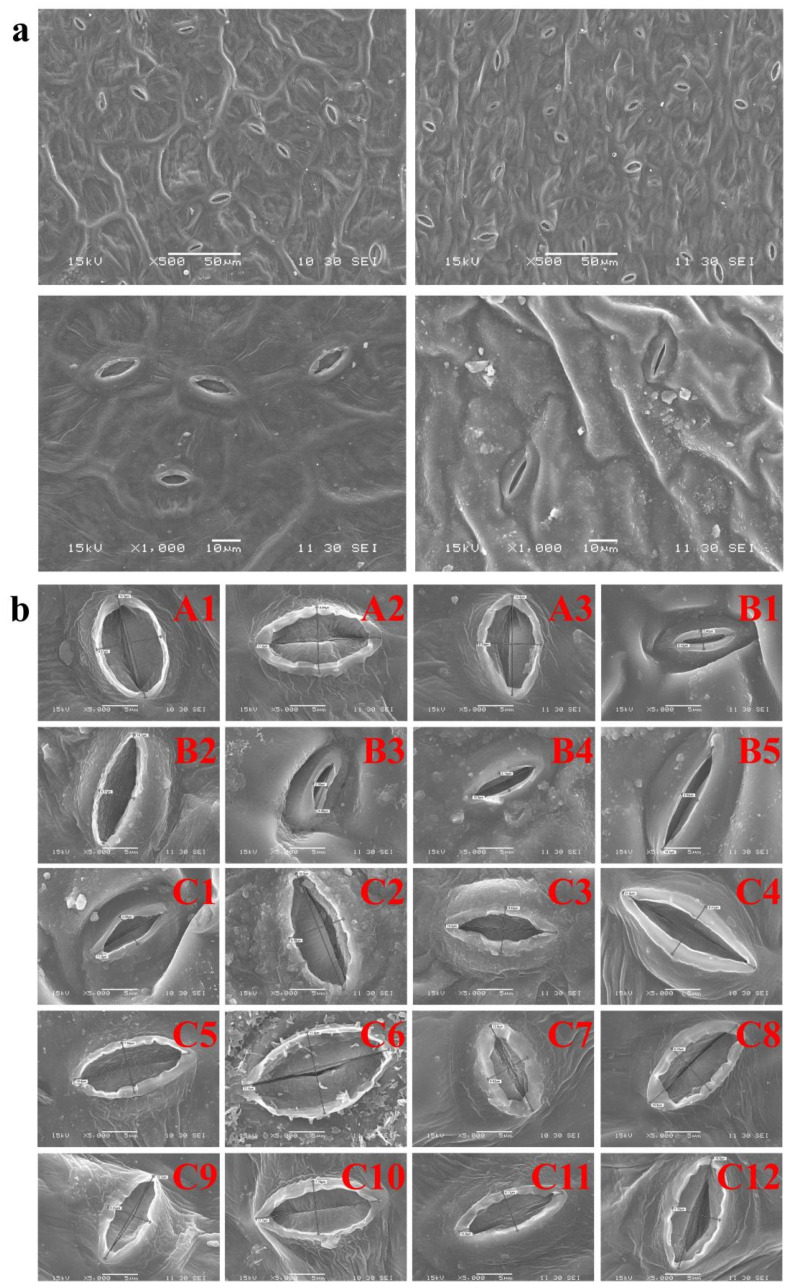
Scanning electron micrographs (SEM) of leaves of 20 leafy vegetables: A, water spinach (*Ipomoea aquatica Forssk*); B, amaranth (*Amaranthus tricolor*); C, cabbage (*Brassica pekinensis*). (**a**) SEM observation of atmospheric particulate matter on leaves under 500× and 1000× magnification. (**b**) Scanning electron microscope of stomata of 20 leafy vegetables under 5000× magnification.

The scanning electron micrographs of the 20 varieties leafy vegetables are shown in Figure 5a,b. Shao et al. (2019) observed particulate matter on the leaf surface through SEM under 500× magnification [45]. This study also used SEM to observe the leaf surface morphology in 20 leafy vegetables. Many atmospheric particles can be obviously seen scattered on the surface of leaves under 500× magnification (Figure 5a). When the magnification was set to 1000×, the particles were observed near the stomata and leaf folds and the particles were smaller than the size of stomata (Figure 5a). Therefore, it can be concluded that leafy vegetables can absorb heavy metals through the foliar uptake of atmospheric particles. Correspondingly, Zhou et al. (2016) and Jolly et al. (2013) reported that leafy vegetables appear to have the highest propensity to accumulate trace elements through investigating TF values of trace elements in 22 vegetable species of six types (leafy, legume, root, stalk, solanaceous, and melon vegetables) [46,47]. In addition, Pb and Cd were likely to concentrate in fine particles (Figure 1), indicating that the heavy metals that were enriched in particles deposited on the leaf surface were more capable of entering the plant through the stomata. Furthermore, Gao et al. (2022) suggested that small particles might diffuse through both stomatal and cuticular pathways to enter plant leaves, being transferring into the vegetables [48]. Many studies confirmed that heavy metals attached to PMs contribute to heavy-metal accumulation, especially Pb accumulation, in leaf vegetables in urban or suburban areas [49]. Therefore, the stomata size should be considered as a crucial index for evaluating the strength of foliar uptake [21].

Bu et al. (2014) used the width-to-length ratio to represent the degree of stomatal opening and closing [25]. This study also showed the degree of stomatal opening and closing by measuring the long and short axis of the stomata (Table S1). The size of stomata of the three species of leafy vegetables exhibited the following trend: water spinach (0.60) > cabbage (0.45) > amaranth (0.29) (Table S1). Moreover, it can be seen that heavy-metal contents were higher in leaves and lower in roots for water spinach species, and the higher accumulation in leaves was consistent with a larger stomatal width-to-length ratio in different varieties, indicating that the significant difference in heavy-metal concentration in leaves might be caused by foliar uptake through the stomata. Similarly, the results were generally consistent in both amaranth and cabbage, except for C1, C2, and C3, of which Pb and Cd were likely transferred from the roots to leaves due to the high concentration in the roots. However, this trend was not reflected in all of the 20 varieties. The contents of Pb and Cd in the three species of leafy vegetables exhibited the following trend: amaranth (1.77 mg kg^−1^) > cabbage (1.38 mg kg^−1^) > water spinach (1.20 mg kg^−1^) for Pb, and cabbage (0.67 mg kg^−1^) > amaranth (0.42 mg kg^−1^) > water spinach (0.26 mg kg^−1^) for Cd (Figure 2 and Figure 3), which is inconsistent with the size of stomata in the leaves. This inconsistency may be due to other foliar morphological characteristics such as leaf inclination, branch density, plant canopy structure, leaf area, etc. [40]. Furthermore, Bi et al. (2018) reported that heavy-metal concentrations in leafy vegetables varied among species [20]. Pan et al. (2016) reported that heavy-metal accumulation in vegetables varied significantly among varieties, and the accumulation of Cd and Cr was highest in *Chicorium endiva* L. and *Spinacia oleracea* L. [50]. Therefore, this experiment also measured leaf areas (Table S2) in an attempt to explain the influencing factors of heavy-metal accumulation in leafy vegetables.

### 4.3. Influencing Factors of Pb and Cd Accumulation in Leafy Vegetables

**Figure 6:**
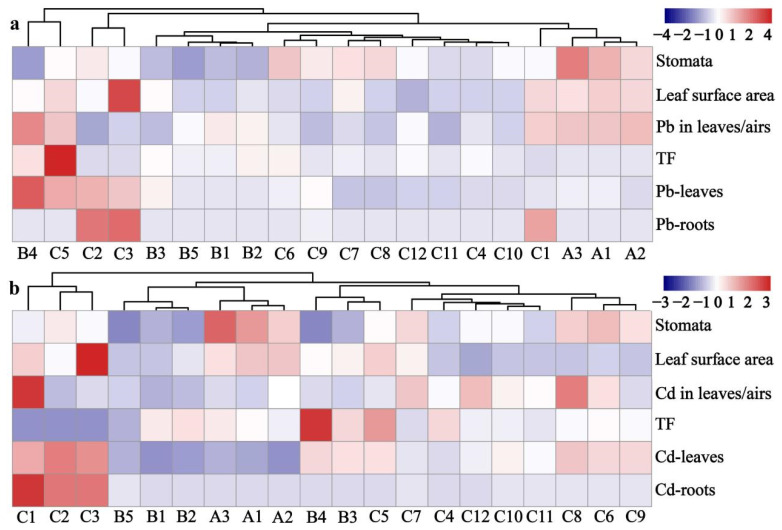
Heat map showing the distribution profiles and physiological and chemical indicators of 20 leafy vegetables ((**a**) Pb, (**b**) Cd).

In order to explore the influencing factors of heavy-metal accumulation in leafy vegetables, stomata width-to-length ratio, leaf area size, EF, and TF were measured. Combined with various indicators, a clustering heat map was generated to illustrate the phenomenon (Figure 6). It can be seen in Figure 6a that B4 and C5 were separated into a single category with high contents of Pb in leaves and low contents in roots. From a further analysis, it was found that they had higher leaf/air values and leaf surface areas, indicating that they might have accumulated heavy metal via relatively strong foliar uptake. Therefore, these two varieties were deemed unsuitable for planting in kitchen gardens and suburban areas where the air might be polluted. In addition, A1, A2, and A3 were separated into a single category characterized as having large stomata and leaf surface area, which induce foliar uptake. Correspondingly, their leavef/air values were relatively high, so they were also deemed unsuitable for planting in these areas. On the other hand, the strength of foliar uptake may also be affected by other factors, such as the morphology and surface area of leaves, chemical and physical characteristics of the cuticle, physico-chemical forms of adsorbed metals, plant habitus, exposure duration, environmental conditions, and gas exchange [40]. It can also be seen in Figure 6b, that C8, C6, and C9 had high contents of Cd in their leaves and low contents in their roots. They were thus separated into a single category and deemed unsuitable for planting in suburban areas.

In addition to foliar uptake, heavy-metal uptake from roots was another major pathway of heavy-metal accumulation in leaves. It can be seen that C2 and C3 in Figure 6a, and C1, C2, and C3 in Figure 6b were classified into one category, which had significantly higher contents in the roots. Gao et al. (2010) reported that the high Cd concentration in *pakchoi* was due to the high transpiration rate, which helped to translocate Cd from roots to leaves [51]. Therefore, as a result of the strong root uptake, C1, C2, and C3 were deemed unsuitable for planting in areas with contaminated soils. Finally, B1, B2, and B5 were classified into one category (Figure 6a,b). They had lower heavy-metal contents in the roots and leaves and smaller stomata, which demonstrated weak ability of heavy-metal accumulation. For this reason, B1, B2, and B5 can be planted in kitchen gardens and suburban areas to maintain food safety. Moreover, C4 and C12 also demonstrated low accumulation of both Pb and Cd and can also be recommended.

## 5. Conclusions

In order to explore the influencing factors of Pb and Cd accumulation in leafy vegetables and screen suitable varieties based on foliar uptake, 20 varieties of leafy vegetables were grown in a micro-area experiment. This study creatively investigated the contribution of foliar uptake to heavy-metal accumulation in leafy vegetables and explored the effect of leaf area and stomata size on heavy-metal accumulation in leafy vegetables. It can be seen that foliar uptake was an important source of Pb and Cd accumulation in leafy vegetables. The study indicated that water spinach accumulated enormous amounts of Pb through foliar uptake, and the amount mainly depended on the size of stomata. B1, B2, B5, C4, and C12 demonstrated a weaker ability to absorb heavy metals through foliar uptake and are recommended to be planted in areas prone to air pollution. Furthermore, as a result of the strong root uptake, C1, C2, and C3 were deemed unsuitable for planting in areas with contaminated soil. The results provide technical support for the safe production of leafy vegetables. However, the various factors that affect foliar uptake are complicated and include leaf inclination, branch density, plant canopy structure, leaf area, and stomata size, etc. Therefore, further studies, including microscopic or quantitative methods, are required to explore the mechanism of foliar uptake and quantify its contribution to heavy-metal accumulation in leaves.

## Figures and Tables

**Table 1:** Three species of twenty common edible vegetables.

Specie	Abbreviation	Genotypes
Water spinach (*Ipomoea aquatica Forssk*)	A1	Baigengliuyekongxincai
	A2	Taiguokongxincai
	A3	Dayekongxincai
Amaranth (*Amaranthus tricolor*)	B1	Yidianhongxiancai
	B2	Qingxiancai
	B3	Hongliuyexiancai
	B4	Qingliuyexiancai
	B5	Baiyuanyexiancai
Cabbage (*Brassica pekinensis*)	C1	Shenyangkuaicai38
	C2	Xinzajiaokuaicai50
	C3	Meiweitiankuaicai
	C4	Nanjingjiangengbai
	C5	Xiangruikuaicai536
	C6	Jimaocai
	C7	Suzhouqing
	C8	Jindiansijiqing
	C9	Baixuegongzhu
	C10	Zaoshutiancaixin
	C11	Choutaiqinggengcai
	C12	Sijixiaobaicai

**Table 2:** The EDD values (mg kg^−1^ day^−1^) of Pb and Cd for adults and children through ingestion of leafy vegetables.

Specie	Vegetable Types	EDD (Pb)		EDD (Cd)	
		Adults	Children	Adults	Children
Water spinach (*Ipomoea aquatica Forssk*)	A1	1.50 × 10^−5^	2.86 × 10^−5^	3.30 × 10^−6^	6.28 × 10^−6^
	A2	1.28 × 10^−5^	2.44 × 10^−5^	2.51 × 10^−6^	4.79 × 10^−6^
	A3	1.60 × 10^−5^	3.05 × 10^−5^	3.70 × 10^−6^	7.04 × 10^−6^
Amaranth (*Amaranthus tricolor*)	B1	1.35 × 10^−5^	2.58 × 10^−5^	2.58 × 10^−6^	4.90 × 10^−6^
	B2	1.35 × 10^−5^	2.58 × 10^−5^	2.91 × 10^−6^	5.54 × 10^−6^
	B3	2.00 × 10^−5^	3.80 × 10^−5^	7.92 × 10^−6^	1.51 × 10^−5^
	B4	4.71 × 10^−5^	8.97 × 10^−5^	8.28 × 10^−6^	1.58 × 10^−5^
	B5	1.37 × 10^−5^	2.61 × 10^−5^	3.81 × 10^−6^	7.25 × 10^−6^
Cabbage (*Brassica pekinensis*)	C1	1.36 × 10^−5^	2.60 × 10^−5^	1.02 × 10^−5^	1.94 × 10^−5^
	C2	3.12 × 10^−5^	5.94 × 10^−5^	1.21 × 10^−5^	2.31 × 10^−5^
	C3	2.80 × 10^−5^	5.34 × 10^−5^	1.12 × 10^−5^	2.13 × 10^−5^
	C4	1.19 × 10^−5^	2.27 × 10^−5^	5.28 × 10^−6^	1.01 × 10^−5^
	C5	3.22 × 10^−5^	6.12 × 10^−5^	7.93 × 10^−6^	1.51 × 10^−5^
	C6	1.59 × 10^−5^	3.03 × 10^−5^	8.45 × 10^−6^	1.61 × 10^−5^
	C7	8.59 × 10^−6^	1.63 × 10^−5^	5.64 × 10^−6^	1.07 × 10^−5^
	C8	8.66 × 10^−6^	1.65 × 10^−5^	8.94 × 10^−6^	1.70 × 10^−5^
	C9	1.86 × 10^−5^	3.54 × 10^−5^	8.40 × 10^−6^	1.60 × 10^−5^
	C10	1.28 × 10^−5^	2.44 × 10^−5^	7.30 × 10^−6^	1.39 × 10^−5^
	C11	1.04 × 10^−5^	1.99 × 10^−5^	6.35 × 10^−6^	1.21 × 10^−5^
	C12	9.84 × 10^−6^	1.87 × 10^−5^	5.92 × 10^−6^	1.13 × 10^−5^

## Supplementary Materials

The following supporting information can be downloaded at: https://www.mdpi.com/article/10.3390/life12030339/s1, Table S1: The stomatal width-to-length ratio of 20 vegetables; Table S2: The Leaf surface area of 20 vegetables.
