# Supplementary material for: Screening of Leafy Vegetable Varieties with Low Lead and Cadmium Accumulation Based on Foliar Uptake
Source: Life (Basel). 2022 Feb 24;12(3):339. doi: 10.3390/life12030339 (PMC8955535; doi:10.3390/life12030339)
Supplement: Supplementary file 1 [file life-12-00339-s001.zip › life-1560198-supplementary.pdf]

Cover sheet for Supplementary Material for Publication

Zhangqian Xu[a], Jianwei Peng[a], Zhen Zhu[a], Pengyue Yu[a], Maodi Wang[a], Zhi Huang[a], Ying Huang[a, b]* Zhaojun Li[b]

[a] National Engineering Research Center for Efficient Utilization of Soil and Fertilizer Resources, College of Resources and Environment, Hunan Agricultural University, Hunan, 410128, China

[b] Institute of Agricultural Resources and Regional Planning, Chinese Academy of Agricultural Sciences, Key Laboratory of Plant Nutrition and Fertilizer, Ministry of Agriculture, Beijing 100081, PR China

Manuscript title: Varieties screening of leafy vegetables with low accumulation of lead and cadmium based on foliar uptake

Number of pages: 3

Number of tables: 2

Table of contents

Table S1. The stomatal width-to-length ratio of 20 vegetables.

Table S2. The Leaf surface area of 20 vegetables.

Table S1. The stomatal width-to-length ratio of 20 vegetables.

Specie	Abbreviation	width-to-length ratio
Water spinach (*Ipomoea aquatica Forssk*)	BGLYKXC (A1)	0.63±0.06
	TGKXC (A2)	0.54±0.05
	DYKXC (A3)	0.65±0.13
Amaranth (*Amaranthus tricolor*)	YDHXC (B1)	0.35±0.04
	QXC (B2)	0.33±0.21
	HLYXC (B3)	0.33±0.15
	QLYXC (B4)	0.31±0.08
	BYYXC (B5)	0.12±0.03
Cabbage (*Brassica pekinensis*)	SYKC-38 (C1)	0.42±0.07
	XZJKC-50 (C2)	0.47±0.18
	MWTKC (C3)	0.43±0.10
	NJJGB (C4)	0.39±0.05
	XRKC-536 (C5)	0.45±0.12
	JMC (C6)	0.55±0.16
	SZQ (C7)	0.49±0.12
	JDSJQ (C8)	0.5±0.12
	BXGZ (C9)	0.48±0.07
	ZSTCQ (C10)	0.44±0.23
	CTQGC (C11)	0.39±0.11
	SJXBC (C12)	0.43±0.03

Table S2. The Leaf surface area of 20 vegetables.

Specie	Abbreviation	Leaf surface area (cm^2)
Water spinach (*Ipomoea aquatica Forssk*)	BGLYKXC (A1)	501.2±0.25
	TGKXC (A2)	474.19±0.15
	DYKXC (A3)	406.03±0.18
Amaranth (*Amaranthus tricolor*)	YDHXC (B1)	108.48±0.15
	QXC (B2)	201.63±0.09
	HLYXC (B3)	321.65±0.21
	QLYXC (B4)	289.49±0.18
	BYYXC (B5)	112.64±0.08
Cabbage (*Brassica pekinensis*)	SYKC-38 (C1)	453.82±0.15
	XZJKC-50 (C2)	283.27±0.09
	MWTKC (C3)	1064.45±0.31
	NJJGB (C4)	104.24±0.09
	XRKC-536 (C5)	447.13±0.12
	JMC (C6)	146.08±0.10
	SZQ (C7)	341.64±0.21
	JDSJQ (C8)	98.55±0.10
	BXGZ (C9)	108.93±0.15
	ZSTCQ (C10)	122.64±0.07
	CTQGC (C11)	101.47±0.18
	SJXBC (C12)	10.16±0.05
